# Quorum Sensing Signal Production and Microbial Interactions in a Polymicrobial Disease of Corals and the Coral Surface Mucopolysaccharide Layer

**DOI:** 10.1371/journal.pone.0108541

**Published:** 2014-09-30

**Authors:** Beth L. Zimmer, Amanda L. May, Chinmayee D. Bhedi, Stephen P. Dearth, Carson W. Prevatte, Zoe Pratte, Shawn R. Campagna, Laurie L. Richardson

**Affiliations:** 1 Department of Biological Sciences, Florida International University, Miami, Florida, United States of America; 2 Atkins North America, Miami, Florida, United States of America; 3 Department of Chemistry, University of Tennessee, Knoxville, Tennessee, United States of America; Universidade Federal do Rio de Janeiro, Brazil

## Abstract

Black band disease (BBD) of corals is a complex polymicrobial disease considered to be a threat to coral reef health, as it can lead to mortality of massive reef-building corals. The BBD community is dominated by gliding, filamentous cyanobacteria with a highly diverse population of heterotrophic bacteria. Microbial interactions such as quorum sensing (QS) and antimicrobial production may be involved in BBD disease pathogenesis. In this study, BBD (whole community) samples, as well as 199 bacterial isolates from BBD, the surface mucopolysaccharide layer (SML) of apparently healthy corals, and SML of apparently healthy areas of BBD-infected corals were screened for the production of acyl homoserine lactones (AHLs) and for autoinducer-2 (AI-2) activity using three bacterial reporter strains. AHLs were detected in all BBD (intact community) samples tested and in cultures of 5.5% of BBD bacterial isolates. Over half of a subset (153) of the isolates were positive for AI-2 activity. AHL-producing isolates were further analyzed using LC-MS/MS to determine AHL chemical structure and the concentration of (*S)*-4,5-dihydroxy-2,3-pentanedione (DPD), the biosynthetic precursor of AI-2. C6-HSL was the most common AHL variant detected, followed by 3OC4-HSL. In addition to QS assays, 342 growth challenges were conducted among a subset of the isolates, with 27% of isolates eliciting growth inhibition and 2% growth stimulation. 24% of BBD isolates elicited growth inhibition as compared to 26% and 32% of the bacteria from the two SML sources. With one exception, only isolates that exhibited AI-2 activity or produced DPD inhibited growth of test strains. These findings demonstrate for the first time that AHLs are present in an active coral disease. It is possible that AI-2 production among BBD and coral SML bacteria may structure the microbial communities of both a polymicrobial infection and the healthy coral microbiome.

## Introduction

**Figure 1:**
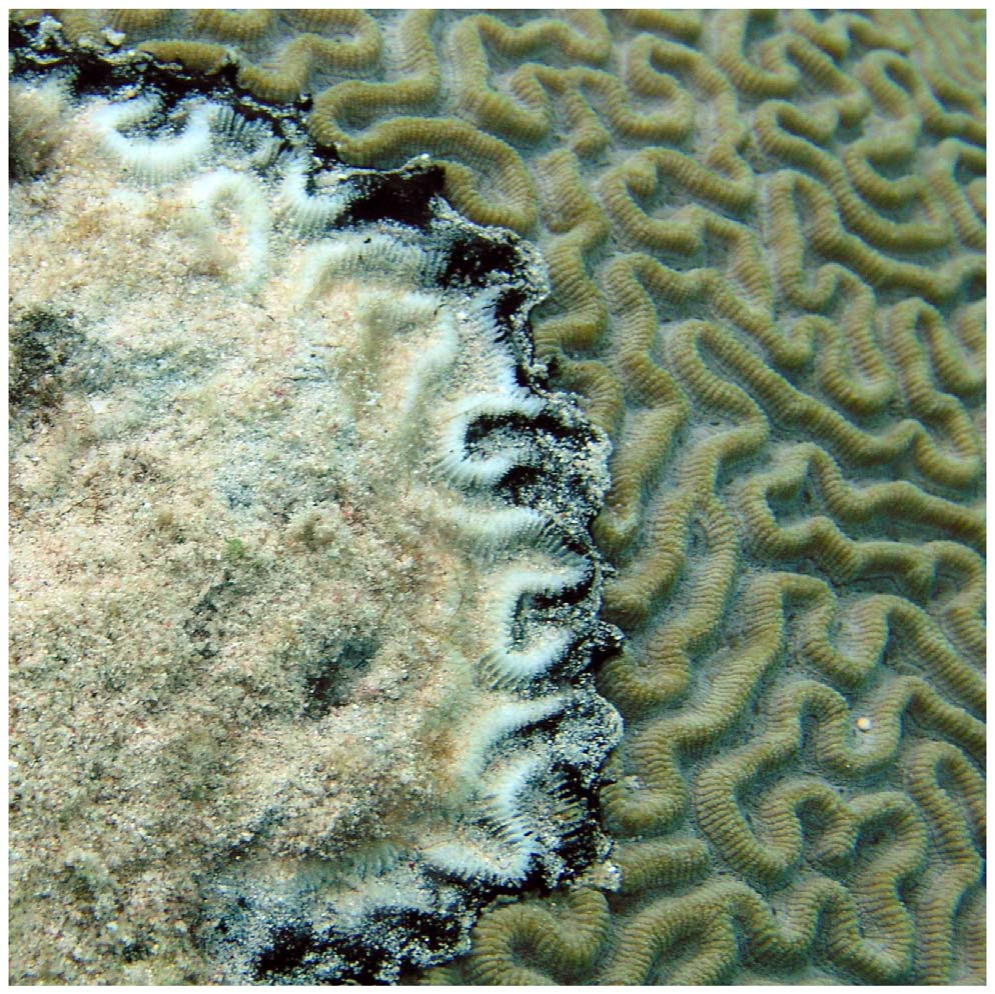
Black band disease infection on a colony of *Diploria strigosa* on a reef of Curaçao. The dark-colored black band disease microbial mat separates apparently healthy coral tissue from white, denuded coral skeleton. Photograph provided by Abigael Brownell.

Coral diseases are widely believed to play a key role in the deterioration of coral reefs on a global basis [1], [2], with black band disease (BBD) identified as one of the major coral diseases contributing to this decline [1], [3]. BBD is easily distinguished in the reef environment (Figure 1), manifesting as a dark-colored band separating healthy coral tissue from recently exposed coral skeleton. The band migrates horizontally across the surface of a coral colony, causing coral tissue necrosis at a rate of approximately 3.0 mm per day [4]. The tissue loss on an individual colony infected with BBD can be substantial and can result in total colony mortality [5], [6], [7]. Massive reef-building corals are susceptible to BBD [2], [4], [8], which exacerbates the impact of the disease on reef ecology and function [6].

The BBD mat consists of a microbial consortium dominated by filamentous cyanobacteria [7], [9], [10], [11], [12], [13]. Mechanisms of BBD pathogenicity include anoxic conditions within the BBD mat in combination with sulfide production by BBD sulfate reducing bacteria [14], and BBD cyanobacterial toxin (microcystin) production [15], [16], [17], [18]. A recent microscopic study [19] documented cellular necrosis (i.e., loss of tissue confluence, cell-to-cell adhesion, cytoplasmic disintegration, nuclear breakdown, and the presence of autophagous bodies, pyknotic nuclei, and apoptotic bodies) in the coral tissue surrounding cyanobacterial filaments in active BBD infections. These microscopic observations support the previous studies demonstrating the role cyanobacterial toxins in BBD pathogenicity.

A meta-analysis of 87 published BBD clone libraries from the Caribbean and Indo-Pacific [20] detected a common cyanobacterial sequence, recently characterized as *Roseofilum reptotaenium* gen. et sp. nov. [21], as present in 78% of the clone libraries. In contrast to the low diversity of BBD cyanobacterial taxa, the meta-analysis also revealed an extremely high diversity of heterotrophic BBD bacteria, with 73% of all sequences detected present as singletons (only 1 copy in the 87 clone libraries). Very little is known about the role of BBD heterotrophic bacteria, the one exception being that BBD has a well-documented and very active sulfur cycle generated by sulfate reducing bacteria within the disease consortium [22], [23].

BBD infections occur on the surface tissues of infected coral colonies, which are also known to be microbially diverse. In particular, the coral surface mucopolysaccharide layer (SML) supports a dynamic microbial community [24], [25] which is believed to play an important role in coral disease resistance [26]. Interactions between several bacterial coral pathogens and the microbial community of the coral SML have been the subject of multiple studies (reviewed in [26]), and the coral SML and its associated microbes have been shown to produce antimicrobial agents and biofilm inhibitors that may be acting to protect corals from pathogen growth [27], [28], [29], [30], [31]. However, relatively little is currently known about the interactions between microbes within the SML.

Chemical signaling in tropical and subtropical coastal environments is a relatively unexplored area that may be important in coral health and disease. Quorum sensing (QS), a density dependent form of bacterial cell-cell communication, is one mechanism by which coral pathogenic bacteria and SML bacteria may be interacting [32]. Acyl-homoserine lactones (AHLs) and autoinducer-2 (AI-2) are two of the more well-characterized groups of QS signaling molecules [33]. The latter (AI-2) is a family of related molecules derived from (*S*)-4,5-dihydroxy-2,3-pentanedione (DPD) [34]. AHLs are considered to be intraspecies signaling molecules [35], although it has been shown that cross-talk between bacteria using these molecules can occur (e.g., [36], [37], [38]). AHL production has been well documented among members of the proteobacteria [39], [40], a group that is commonly detected in BBD clone libraries [10], [13], [41], [42], [43], making AHL production a prime target for BBD research. AI-2 signaling is widely recognized as having an important role in interspecies communication [35], [44], [45], [46], since DPD production is common in both Gram-negative and Gram-positive bacteria [47], [48]. AI-2 signaling may also play a role in the complex bacterial communities of both BBD and coral SML. Overall, QS is associated with a wide range of interactive social responses in bacteria (see [44], [49]) and has been shown to regulate virulence in both Gram-positive and Gram-negative pathogens [35], including upregulation of antibiotic biosynthesis [50], [51]. AHL production has been observed in coral-associated bacteria isolated from the SML of healthy corals [52], isolates from other marine invertebrates and their endosymbiotic dinoflagellates [27], and from the tissues of 10 cnidarian species (all healthy) examined recently [53]. *Vibrio* spp. isolated from the mucus of healthy and diseased corals have been shown to produce both AHLs and DPD [54]. In one study, QS was proposed to play a major role in the pathogenicity of the coral pathogen *Vibrio coralliitycus* [55]. With the exception of the study presented here, the *in situ* presence of QS signals in active coral disease has not yet been demonstrated. The roles of these signals in the coral microbial community and in coral disease remain unknown.

## Materials and Methods

### Field sampling

Black band disease and coral mucus samples were collected from reefs in Ft. Lauderdale, Florida under Florida Fish and Wildlife Conservation Commission sampling license SAL-11-1344-SRP and from Florida Keys reefs under permits FKNMS-2007-026 and FKNMS 2009-045-A3. Freshly collected BBD mat as well as both bacterial (N = 191) and cyanobacterial (N = 8) isolates from BBD and coral mucus samples were examined for production of autoinducers in this study (Table 1). The bacterial isolates included 182 newly isolated strains cultured from colonies of *Montastraea cavernosa, Colpophyllia natans,* and *Diploria strigosa* located on the Florida Reef Tract [Ft. Lauderdale, Florida, USA (26° 11.35′ N, 80° 5.49′ W); Horseshoe Reef, Florida Keys, USA (25° 08.362′ N, 80° 17.641′); and Algae Reef, Florida Keys, USA (25° 08.799′ N, 80° 17.579′ W)] (Table 1). Thirty-nine of the 191 bacteria were newly isolated from *Diploria strigosa* on a reef of Curaçao (Water Factory 3, 12° 06.779′ N, 68° 57.662′ W). Samples were collected using sterile, needleless 10 ml or 60 ml syringes. SML from apparently healthy areas of BBD-infected colonies (designated BSML) was sampled at the farthest distance (minimum of 20 cm) from the BBD mat. SML samples were also collected from apparently healthy colonies (HSML) of the same coral species located in the near vicinity of BBD-infected corals. To collect SML, the coral surface was gently agitated using the syringe tip (to cause mucus secretion) and the resulting mucus aspirated into the syringe. The BBD mat was easily collected by syringe as it is loosely attached to the coral surface.

**Table 1:** Sampling and bacterial isolate designations.

Site Name and Location	Date ofCollection	WaterDepth (m)	WaterTemp (°C)	Colony No.	Host Coral	SampleType(s)^1^	Bacterial IsolateDesignations^2^^,^ 3 ^,^ 4	No. of IsolatesExamined
Ft. Lauderdale, Broward County, FL, USA; 26° 11.35′ N, 80° 5.49′ W	17-May-08	3.7	28	FTL 1	*Montastraea cavernosa*	BBD	BBD-FTL-1x	109 (41 BBD, 23 BSML, 45 HSML)
	27-Jun-08	4.6	28	FTL 2	*M. cavernosa*	BBD	BBD-FTL-2x	
	27-Jun-08	4.3	28	FTL 3	*M. cavernosa*	BBD	BBD-FTL-3x	
	27-Jun-08	4.0	28	FTL 4	*M. cavernosa*	BBD	BBD-FTL-4x	
	27-Jun-08	3.3	28	FTL 5	*M. cavernosa*	BBD	BBD-FTL-5x	
	1-Aug-09	4.6	29	FTL 6	*M. cavernosa*	BBD, BSML	BBD-FTL-6x, BSML-FTL-6x	
	31-Jul-10	3.7	30	FTL 7	*M. cavernosa*	BBD, BSML	BBD-FTL-7x, BSML-FTL-7x	
	31-Jul-10	4.3	30	FTL 8	*M. cavernosa*	BBD, BSML	BBD-FTL-8x, BSML-FTL-8x	
	31-Jul-10	4.6	30	FTL 9	*Diploria strigosa*	HSML	HSML- FTL-9x	
	31-Jul-10	4.3	30	FTL 10	*M. cavernosa*	HSML	HSML- FTL-10x	
	31-Jul-10	4.3	30	FTL 11	*M. cavernosa*	HSML	HSML- FTL-11x	
Algae Reef, Florida Keys, FL, USA; 25° 08.799′ N, 80° 17.579′ W	24-Oct-09	6.7	25	FLK 1	*D. strigosa*	BBD, BSML	BBD-FLK-1x, BSML-FLK-1x^5^	27 (13 BBD, 14 BSML)
Horseshoe Reef, Florida Keys, FL, USA; 25° 08.362′ N, 80° 17.641′W	7-Sept-12, 30-Sept-12	3.7	29	FLK 2^6^	*Colpophyllia natans*	BBD	BBD-FLK-1Mx, BBD-FLK-1Sx,BBD-FLK-2Mx, BBD-FLK-2Sx	7
Water Factory 3, Curacao; 12° 06.779′ N, 68° 57.662′ W	23-Feb-13	2.5	27	CUR 1	*D. strigosa*	BBD	BBD-CUR-3Mx, BBD-CUR-3Sx	39
Horseshoe Reef, Lee Stocking Island, Bahamas; 23° 46.30′ N, 76° 5.33′ W	9-Jul-04	8.2	29	216	*Siderastrea* *siderea*	BBD	BBD-216-x	5
		7.3	28	217	*S. siderea*	BBD	BBD-217-x	4

1 BBD samples were obtained from a BBD mat sample; BSML samples were obtained from the SML of an apparently healthy area of a BBD-infected colony; HSML samples were obtained from the SML of an apparently healthy colony.

2 The “x” in the isolate designation refers to an individual isolate from the source colony (e.g., BBD-FTL-1a, BBD-FTL-1b, etc.).

3 The “M” in the isolate designation refers to isolates that were selected from a marine agar plate.

4 The “S” in the isolate designation refers to isolates that were selected from a sea water tryptone agar plate.

5 Includes BBD-216-1b, BBD-216-2d, BBD-216-3d, BBD-216-4a, BBD-216-4e, BBD-217-2b, BBD-217-2d, BBD-217-2g, and BBD-217-3m. Sampling and identification information available from [42], [43].

6 Two BBD samples were collected from the single colony of *C. natans* on separate days. Sample 1 and Sample 2 were collected on 27-Sept-12 and 30-Sept-12, respectively.

Syringes with freshly collected samples were held in coolers at ambient seawater temperature during transport to the laboratory, where the SML sample syringes were inverted to allow sampled mucus to settle to the syringe tip for collection. BBD samples clumped within the syringe shortly after aspiration (a behavioral response of the filamentous cyanobacteria) and each clump was directly collected and transferred into sterile 2.0 ml cryovial. Each SML sample was extruded into a sterile 2.0 ml cryovial to limit the amount of seawater in the sample. Autoclaved artificial seawater was then added to each cryovial. Samples were mixed by vortexing and spread plated, after a standard dilution series, onto plates containing the following media: Difco marine agar (MA), 1/10 strength MA, Thiosulfate Citrate Bile Salts Sucrose (TCBS; *Vibrio*-specific, BD) agar, and Sea Water Tryptone (SWT) agar. Plates were incubated at room temperature (∼23°C) and unique colonies were selected on the basis of color and morphology for further isolation and evaluation.

In addition to the above isolates, 17 previously isolated BBD bacteria (N = 9) and cyanobacteria (N = 8), collected in the Florida Keys, Bahamas, and Philippines were tested for QS signal production (Table 1). The nine bacterial cultures (strains BBD-216-1b, BBD-216-2d, BBD-216-3d, BBD-216-4a, BBD-216-4e, BBD-217-2b, BBD-217-2d, BBD-217-2g, and BBD-217-3m, all documented in [42], [43]) were maintained on MA slants at room temperature. The eight cyanobacterial cultures (strains BBD 1991, HS 217, HS 223, W-1, FLK BBD1, Phil 2b-2, 102a-1, and 96-2) are all members of the genera *Geitlerinema* and *Leptolyngbya* and were collected on various reefs in the Florida Keys, Bahamas, and the Philippines. Information about these isolates is detailed in [8], [12], [15], [17], [56], [57]. The cyanobacterial cultures were maintained in 125 ml Erlenmeyer flasks in algal mineral media ASNIII or marine BG11 at 26°C under a 12∶12 h light:dark cool white fluorescent light regime at an intensity of 20 µE m−2 s−1.

### Cell-Free Culture Filtrates

Cell-free culture filtrates (CFs) from the bacterial isolates were collected for use in QS assays. Isolates were grown in sterile Difco marine broth (MB) that was filtered twice (Whatman 1 paper filters, 5.5 cm) to remove the medium precipitate. The cultures were grown to stationary phase at 29°C with shaking. This temperature is ecologically relevant, since BBD is most active in the wider Caribbean when surface water temperatures are above approximately 28°C [8], [58], [59], [60], [61]. Bacterial cell concentrations were monitored by measuring the optical density at 600 nm (OD_600_) using either a Modulus Microplate Multimode Reader (Turner BioSystems, Sunnyvale, CA, USA) or a Thermo UV1 Spectrophotometer (Thermo Electron Ltd., Cambridge, UK) with sterile MB as a zero/blank.

At early stationary phase, CF samples were prepared by centrifugation at 12,000 *g* for 10 min, and the supernatant was divided into two, 1.0 ml aliquots. Because alkaline conditions have been shown to result in AHL lactonolysis [62], [63], [64], prior to division of the sample the pH of the supernatant was measured using a Jenco Model 60 Digital pH meter (Jenco Electronics, Ltd., Taipei, Taiwan). Since AHLs have been shown to remain stable for extended time periods at pH 5.0–6.0 [65], the pH of the supernatant to be used in the AHL assays was adjusted to this pH range using a sterile HCl solution (1 N). Each CF was then filter sterilized (0.22 µm membrane, Millipore, Billerica, MA, USA) and the CFs were stored at −20°C. For the cyanobacterial strains, CFs were prepared by obtaining a 2.0 ml aliquot from an active cyanobacterial culture. The culture was vortexed and cells were removed using a combination of centrifugation (12,000 *g* for 10 min) and filter sterilization. The cyanobacterial CFs were used immediately in the QS assays (with no acidification).

### AHL Reporter Strain Assays

*Chromobacterium violaceum* CV026 and *Agrobacterium tumefaciens* NTL4(pZLR4) reporter strains were used to detect the presence of short-chain and medium- to long-chain AHLs, as indicated in Table 2. The *C. violaceum* CV026 assay used in this study follows the protocol of [66] with slight modification. *C. violaceum* CV026 was cultured in sterile Difco Luria-Bertani (LB) broth overnight at 30°C and used to prepare the assay plates. The assay plates (triplicates) contained a base of 1.5% Difco LB agar with an overlay of 100 µl of the *C. violaceum* CV026 culture (OD_600_ = 1.0) in 5.0 ml of 0.7% LB agar. Samples tested for the presence of short-chain AHLs consisted of both CFs and biomass (colonies from a plate) of the 199 bacterial and cyanobacterial isolates, as well as freshly collected (full community) BBD mat (Table 3). To test the bacterial CFs, wells were punched in the assay plates using the wide end of a sterile pipette tip. To each experimental well, 75 µl of the appropriate CF was added. To test the BBD mat material, a portion of each BBD sample was placed in 1.0 ml of sterile seawater. The mixture was shaken on a vortexer and 75 µl of the solution was placed into an experimental well in each assay plate. Each assay plate contained a positive control (i.e., 0.01 µl of 50 mM *N*-hexanoyl-L-homoserine lactone spotted on the agar surface) and a negative control (i.e., a well containing 75 µl of sterile MB for the CF assays or a well containing 75 µl of sterile seawater for the BBD mat assays). The assay plates were incubated for 24 h at 30°C and then examined for purple coloration (violacein) surrounding the wells which indicated a positive result. For the bacterial isolate patch tests, each isolate was streaked onto a plate containing MA and incubated at 29°C until colonies were visible. Colonies were collected using a sterile loop and transferred to the surface of *C. violaceum* CV026 assay plates. In the case of the cyanobacterial strains, clumps of cyanobacteria were selected from the culture flask using sterile forceps and transferred to the surface of the assay plates. Each experiment was conducted in triplicate with positive and negative controls, incubated for 24 h at 30°C, and then assessed for violacein presence.

**Table 2:** Quorum sensing reporter strains and positive control strains used in this study.

Name	Strain characteristics	Reference
*Chromobacterium violaceum* CV026^1^	Detects short-chain AHLs (i.e., C4-AHL, C6-3-oxo-AHL, C8-AHL, C8-3-oxo-AHL)	[66]
*Agrobacterium tumefaciens*NTL4(pZLR4)^2^	Detects medium- to long-chain AHLs (i.e., C6-AHL, C10-AHL, C12-AHL, C14-AHL, C6-3-hydroxy-AHL, C8-3-hydroxy-AHL, C10-3-hydroxy-AHL, all 3-oxo-AHLs)	[112]
*Agrobacterium tumefaciens*NT1(pTiC58ΔaccR)^2^	Synthesizes C8-3-oxo-AHL which is recognized by *A. tumefaciens* NTL4(pZLR4). Culture filtrates of this strain were used as positive control in the *A. tumefaciens* NTL4(pZLR4) assay	[113]
*Vibrio harveyi* BB170^3^	Detects the DPD and the CAI-1^4^ signals	[114], [115]
*Vibrio harveyi* BB152^3^	Synthesizes the DPD signal. Culture filtrates of this strain were used as positive control in the DPD assay.	[116]

1 Strain obtained from K. Mathee, Florida International University.

2 Strains obtained from S.K. Farrand, University of Illinois.

3 *V. harveyi* strains BB170 (ATCC BAA-1117) and BB152 (ATCC BAA-1119) obtained from the American Type Culture Collection.

4 CAI-1 is the *Vibrio*-specific QS signal (S)-3-hydroxytridecan-4-one [117].

**Table 3:** Quorum sensing assays conducted in this study.

		*Chromobacterium violaceum CV026 Assay* 1			*Agrobacterium tumefaciens* NTL4(pZLR4) Assay^1^		*Vibrio harveyi* BB170 Assay^1^	
Site Name and Location	No. of IsolatesExamined	PatchTest	CFTest	BBD MatTest	PatchTest	CFTest	BBD MatTest	CulturedIsolates
Ft. Lauderdale	109	+	+	+	+	+	+	+
Algae Reef	27	+	+	+	+	+	+	+
Horseshoe Reef, Florida Keys	7	+	NT	NT	NT	NT	NT	NT
Water Factory 3, Curacao	39	+	NT	NT	NT	NT	NT	NT
Horseshoe Reef, Lee Stocking Island	9	+	+	+	+	+	+	+
BBD cyanobacteria	8	+	+	+	+	+	+	+

1 “+” indicates that the assay was conducted; NT indicates that the assay was not conducted. Patch Test and CF Test refer to isolates and BBD Mat Test refers to intact, freshly collected BBD mat, all tested using the CV026 and NTL4 reporter strains. The BB170 assay utilized actively growing cultures of isolates only (see methods section).

The *Agrobacterium tumefaciens* NTL4(pZLR4) assay used in this study follows the protocol of [67] with slight modification. *A. tumefaciens* NTL4(pZLR4) from frozen glycerol stock was streaked on a plate containing autoinducer bioassay minimal (AB_At_) agar (i.e., 3 g/L K_2_HPO_4_, 1 g/L NaH_2_PO_4_, 1 g/L NH_4_Cl, 0.3 g/L MgSO_4_·7H_2_O, 0.15 g/L KCl, 0.01 g/L CaCl_2_, 0.0025 g/L FeSO_4_·7H_2_O; [68]), along with 5 g/L mannitol (0.5%) and gentamicin (30 µg/ml) and grown at 28°C until colonies were visible. A single colony was transferred via sterile loop to 1.0 ml of AB_At_ medium with gentamicin (30 µg/ml) and grown overnight at 28°C with shaking. The day of the assay, a fresh solution of AB_At_ medium with gentamicin (3 µg/ml) was prepared, inoculated with 50 µl of the overnight culture, and grown to late exponential phase at 28°C with shaking. The assay plates contained a base of 1.5% AB_At_ agar (0.5% mannitol) with an overlay that included 500 µl of the *A. tumefaciens* NTL4(pZLR4) culture in 5.0 ml of 0.7% water agar with gentamicin (30 µg/ml) and 5-bromo-4-chloro-3-indolyl-b-D-galactopyranoside (X-gal; 40 µg/ml). As above (for the *C. violaceum* CV026 reporter strain), assay plates were used to test both CFs and biomass (colonies) of the 199 bacterial isolates, as well as freshly collected BBD mat (Table 3). As a positive control, *A. tumefaciens* NTL4(pTiC58Δ*accR*), which produces C8-3-oxo-AHL, was grown in AB_At_ broth at 28°C with shaking. An aliquot of the culture was centrifuged at 12,000 *g* for 10 min, and the resulting supernatant was filter sterilized. The negative control well on each plate contained either 75 µl of sterile MB for the CF assays or 75 µl of sterile seawater for the BBD mat assays. The assay plates were incubated for 24 h at 28°C and then assessed for the presence of blue coloration (X-gal cleavage) surrounding the wells, which would indicate a potential positive result. To detect if any of the isolates produced an extracellular factor that hydrolyzes X-Gal, isolates that produced a blue coloration in the patch test were retested on a plate containing only AB_At_ agar (0.5% mannitol) with X-Gal (40 µg/L). Isolates producing blue coloration on these plates were considered a false-positive result in the *A. tumefaciens* NTL4(pZLR4) assay and the result was scored as negative.

AHLs were extracted from those isolates that tested positive in at least one AHL reporter strain assay. Extracted AHLs were then analyzed using liquid chromatography–tandem mass spectrometric (LC-MS/MS), which allows for the determination of the AHL chemical structure. The extraction and LC-MS/MS methods are described below.

### AI-2 Reporter Strain Assay

The *V. harveyi* BB170 reporter strain was used to screen for the presence of AI-2 (Table 2) in 153 bacterial CFs (Table 3). The AI-2 assay used in this study follows the protocol developed by [69] with slight modification. A modified autoinducer bioassay (AB_Vh_) medium was prepared (1 L solution of 0.3 M NaCl, 0.05 M MgSO_4_, and 0.2% casamino acids) and adjusted to pH 7.5. This solution was autoclaved (121°C) and the following components were added to the cooled solution from sterile stocks: 0.05 M K_2_HPO_4_ (pH 7.0), 0.001 M L-arginine, and glycerol (to 1.0%). Fresh AB_Vh_ medium was inoculated with an aliquot of the *V. harveyi* BB170 frozen glycerol culture, which was then grown (30°C with aeration, shaking) until turbid and showing obvious luminescence in a darkroom. This culture was then diluted 1∶5,000 in fresh AB_Vh_ medium and used to prepare the assay plates. The CF from the *V. harveyi* BB152 mutant strain served as a positive control in the AI-2 assay (Table 2). *V. harveyi* BB152 was cultured overnight in AB_Vh_ medium (30°C with aeration, shaking), centrifuged (12,000 *g,* 10 min), and the supernatant was filter sterilized and stored at −20°C. AI-2 assays were conducted in sterile 96-well microtiter plates (BD Falcon 353219 - black plate with a clear, flat bottom and lid). Each assay plate contained triplicate experimental wells, control wells, and reference wells, all with final well volumes of 100 µl. The prepared plates were incubated at 30°C with shaking over the course of the 7 h assay. Optical density (OD_600_) and luminescence (490 nm) readings were conducted every 15 min using a BioTek Synergy HT Multi-Mode Microplate Reader linked directly to a PC with Gen5 software (BioTek Instruments, Inc., Winooski, VT, USA). Because some growth media components have been shown to induce luminescence in the *V. harveyi* BB170 reporter strain (e.g., borate and glucose [70], [71], [72]), medium control wells were included to monitor possible growth media effects on reporter strain luminescence. Media reference wells were monitored during the AI-2 assays to determine background luminescence output for each of the media tested.

AI-2 activity was determined at the optimal time point (OTP) of the *V. harveyi* BB170 assay, defined as the time point immediately preceding self-induction by the *V. harveyi* BB170 reporter strain [69]. The OTP represents the time when the mean luminescence of the medium control wells was lowest during the course of the assay. The percentage of AI-2 (% AI-2) activity is expressed as a percentage of the positive control luminescence at the OTP [73]. The % AI-2 activity was calculated as the fold induction of the sample, i.e., the fold change between the sample luminescence and the corresponding reference luminescence (MB for the bacterial CFs and BG11 for the cyanobacterial CFs) divided by the fold induction of the positive control (the fold change between the positive control luminescence and the corresponding reference luminescence, which was AB_Vh_ medium for the positive control CF). The luminescence at the OTP for each sample was used to calculate the induction of luminescence, which is expressed as a fold induction of the sample in comparison to the luminescence of the positive control. The induction of luminescence was calculated by dividing the sample luminescence by the positive control luminescence at the OTP [69], [74].

### AHL Extraction from Isolates in Marine Broth Cultures

Fresh 25 ml aliquots of half strength MB in 125 ml Erlenmeyer flasks were inoculated with 500 µl of cultures grown overnight in the same medium. Duplicate cultures were then grown at 25°C for 24 h with shaking (200 rpm). At this point, 10 ml aliquots of each culture were filtered through 0.22 µm nylon filters (GE Magna) in duplicate to generate a total of four samples per isolate. The filtrate was transferred to a separatory funnel, and the flask was washed with ∼1 ml water and added to the funnel. The filtrate was then extracted twice with 5 ml of 1.0% acetic acid in ethyl acetate (H^+^/EtOAc). The combined organic layers were then dried with MgSO_4_ and filtered. The filtrate was concentrated *in vacuo* and the resulting oil was then resuspended in 300 µl of H^+^/EtOAc and transferred to an autosampler vial. Samples were immediately analyzed via LC-MS/MS [75].

### AHL Extraction from Isolates on Marine Agar Plates

Fresh 25 ml aliquots of half strength MB in 125 ml Erlenmeyer flasks were inoculated with 500 µl of cultures grown overnight in the same medium. Triplicate cultures were then shaken at 200 rpm at 25°C for 1 h. At this time, a 5.0 ml aliquot of each culture was filtered through a 0.45 µm nylon filter (GE Magna), which was placed cell side up on half strength MA plates and incubated at 25°C for 24 h, after which the filter was removed and the agar was minced and transferred to a 250 ml Erlenmeyer flask. A 100 ml aliquot of ethyl acetate (EtOAc) was added to each flask, and the suspension was stirred for 3 h. Once extraction was complete, the organic layer was collected. The flask was rinsed once more with 5.0 ml EtOAc, which was added to the previous organic layer. The combined organic fractions were then dried *in vacuo* and the resulting oils were redissolved in 300 µl of EtOAc, transferred to an autosampler vial, and immediately analyzed via LC-MS/MS [75].

### LC-MS/MS Analysis of AHLs

Analysis of extracted AHLs was performed using an LC-MS/MS method optimized for AHL detection [75]. Samples were kept at 4°C before injection, and 10 µl of each was injected onto a reverse-phase C18 core-shell column (Phenomenex Kinetex, Torrance, CA, USA) via a Thermo Electron Surveyor autosampler (Thermo Fisher Scientific, Waltham, MA, USA). Separation was obtained using a gradient of 0.1% acetic acid in water and 0.1% acetic acid in acetonitrile at a flow rate of 200 µl/min. The eluent was introduced into a TSQ Quantum Ultra Triple Stage Quadrupole mass spectrometer (Thermo Scientific) using electrospray ionization, and detection was achieved using multiple reaction monitoring (MRM) in positive ion mode. This method screens for 54 unique parent *m/z*–fragment *m/z* pairs and relies on the neutral loss of the acyl chain to give a reproducible and characteristic 102 *m/z* fragment to confirm that the molecule was an AHL. All possible chain lengths ranging from 4 to 20 carbons, including both even and odd numbers of carbons, were included in the method, and the potential to have a hydroxyl or ketone at the 3 position, with or without a single double bond in the chain, was also taken into account. Note: this method is unable to distinguish between potential AHL structures that have the same parent m/z-fragment m/z pairs (e.g., 3OC6-HSL and C7-HSL). Therefore, the AHLs were named according to their most likely identity based on reported precedence, e.g., odd chain AHLs are rare and the previous example would be listed as 3OC6-HSL.

The RAW files collected from the instrument were converted to .mzML files using MSconvert from Proteowizard [76] and then imported into MAVEN an open-source software program for interactive processing of LC-MS/MS data that detects and reports peak intensities to produce an extracted ion chromatogram (EIC) for each parent *m/z* – fragment *m/z* determined by the user [77]. An EIC extraction window of 200 ppm was used to visualize the peaks, and the areas for each AHL were integrated. The relative percentage of each detected AHL was calculated from the areas.

### DPD Quantitation from Isolates in Marine Broth Culture

DPD analysis was performed as previously described with slight modification [78]. Briefly, duplicate 300 µl aliquots of culture supernatants were transferred to 1.5 ml centrifuge tubes containing 10 µl of a ^13^C-labeled DPD (^13^C-DPD) internal standard, prepared according to [79] (final internal standard concentration of 500 nM). Each tube was mixed thoroughly by vortexing for 5 s and then centrifuged for 1.5 min at 16.1 rcf to pellet cells. The supernatants (260 µl) were transferred to new 1.5 µl centrifuge tubes containing 25 µl of a 5 mg/ml DPD derivatizing solution [78]. The contents were thoroughly mixed by vortexing and allowed to react for 45 to 60 min. The resulting solutions were then extracted twice with 130 µl of EtOAc and the combined extracts were transferred to 300 µl autosampler vials. The samples were kept at 4°C until LC-MS/MS analysis.

### LC-MS/MS Analysis of DPD

Samples were placed in an autosampler tray cooled to 4°C and 10 µl of each was injected onto a Kinetex 5 µ C18 100 Å 100 × 2.10 mm column. Separation was performed with an isocratic gradient of 95% 0.1% acetic acid in HPLC grade water and 5% HPLC grade Acetonitrile with a flow rate of 200 µl/min for 4 min. The eluent was introduced into a TSQ Quantum Ultra Triple Stage Quadrupole mass spectrometer (Thermo Scientific) using electrospray ionization, and a positive mode selected reaction monitoring protocol was used for detection [78]. The parent *m/z*–fragment *m/z* pairings used for endogenous DPD and ^13^C-DPD were 381 *m/z*–202 *m/z* and 382 *m/z*–203 *m/z*, respectively. A collision energy of 43 was used for both.

The peaks were automatically integrated with manual adjustments using the Xcalibur software (Thermo Electron Corporation). A correction factor was applied to account for natural isotopes [78], and DPD concentration was then calculated by comparing the peak areas of endogenous DPD to the peak areas of the ^13^C-DPD and multiplying by the internal standard concentration.

### 16S rRNA Gene Sequencing

Genomic DNA was extracted from those isolates that tested positive for AHL production using at least one of the reporter strains assays either by placing a single colony in 100 µl of sterile phosphate buffered saline (PBS), heating to 99°C for 10 min, and centrifuging at 5,000 *g* for 10 min, or alternatively by bead-beating using the FastDNA Spin Kit for Soil (Qbiogene, Vista, CA, USA) according to manufacturer’s protocol. DNA was then PCR-amplified using the universal bacterial primers 27F 5′-AGA GTT TGA TCM TGG CTC AG-3′ and 1492R 5′-TAC GGY TAC CTT GTT ACG ACT T-3′ ([80]; Integrated DNA technologies, Coralville, IA, USA). The final concentration for PCR reactions was 1x PCR Buffer, 2.5 mM MgCl_2_, 0.25 mM of each deoxynucleotide triphosphate, 0.5 µM of each forward and reverse primer, 0.25 U GoTaq Flexi DNA Polymerase (Promega), 0.1% bovine serum albumin, DNA-grade sterile water, and 1–2 ng genomic DNA. PCR was carried out in a Peltier Thermal Cycler (PTC-200, MJ Research, Waltham, MA, USA) under the following conditions: 94°C for 3 min, followed by 28 cycles of 94°C for 1 min, 55°C for 30 s, and 72°C for 30 s, with a final soak at 72°C for 20 min. The amplified bacterial 16S rDNA was cleaned using an ExoSAP-IT PCR cleanup kit (USB Corp., Cleveland, OH, USA) and sequenced with an ABI Prism 3100 genetic analyzer (Applied Biosystems, Foster City, CA, USA) at the DNA Core Facility at Florida International University using the BigDye Terminator version 3.1 (Applied Biosystems) with the 27F and 1492R primers. The sequences were trimmed, cleaned, aligned, and assembled using DNA Baser Sequence Assembler (v3.2.5). The nearly full-length sequences (covering the V1–V9 variable regions of the 16S rRNA gene) were then analyzed using the BLAST queuing system [81] to identify their closest relatives in NCBI GenBank. Sequences were submitted to the GenBank database under accession numbers KF494426, KF494427, and KF148595-KF148603.

### Growth Challenge Assays

To determine whether a subset of BBD and SML isolates produce secondary metabolites that impact the growth of other BBD and SML bacteria, CFs from 19 isolates were used to challenge the growth of the other members of this group of isolates. The 19 isolates consisted of nine that tested positive for AHL in at least one of the *C. violaceum* CV026 and *A. tumefaciens* NTL4(pZLR4) reporter strain assays, and an additional 10 isolates that were randomly selected from the pool of isolates that tested negative in both assays. These isolates were also examined for synthesis of DPD. A total of 342 growth challenges was conducted (i.e., each of the 19 CFs were tested against the 18 other isolate cultures). The bacterial growth challenges were conducted in sterile 96-well microtiter plates (BD Falcon 351172). Each isolate was grown in sterile MB overnight at 29°C (shaking) and added to the appropriate wells. Assay plates contained triplicate experimental wells, control wells, and blank wells, with a single reference well for each CF tested to ensure that the CFs were sterile. The final volume in each well was 100 µL. Plates were incubated at 29°C (shaking) and OD_600_ readings were conducted every 30–60 min using the Modulus Microplate Multimode Reader until the bacterial isolate culture control reached stationary phase. The mean growth rate constant and mean generation time were calculated over the linear portion of the growth curve for the untreated control culture and the experimental treatments. Only statistically significant effects on culture growth will be discussed (t-test, p<0.05).

## Results

### AHL Production Detected using Reporter Strains

Nine BBD mat (full community) samples, collected from two Caribbean coral species (*Montastraea cavernosa* and *Diploria strigosa*), were examined using both the *C. violaceum* CV026 and *A. tumefaciens* NTL4(pZLR4) patch test assays. Each test was positive, revealing that both short-chain and medium- to long-chain AHLs are present in freshly collected, active BBD mat.

A total of 199 bacterial isolates (BBD and SML) were examined using the *C. violaceum* CV026 assay. Of these, four (2.0%) tested positive for the patch test and none for the CF test (Table 4). A subset of isolates (N = 153) was also examined using the *A. tumefaciens* NTL4(pZLR4) assay and of these, nine (5.9%) tested positive for either the patch or CF test (Table 4). In total, 11 (5.5%) of the 199 isolates strains were positive for at least one of the two AHL reporter strain assays, and two of these isolates (i.e., BBD-FTL-6j and BBD-FTL-8c) tested positive in both assays. These two isolates were both positive in the patch test with the *C. violaceum* CV026 assay and the CF test with *A. tumefaciens* NTL4(pZLR4), indicating that they produce short-chain AHLs when grown in benthic form (on MA) and medium- to long-chain AHLs when grown in planktonic form (in MB). The two isolates that tested positive for both assays were each a 99% (16S rRNA gene) sequence match in GenBank to one strain of *Vibrio rotiferianus*; however, they were obtained from BBD infections on two separate coral colonies and exhibited different colony morphologies when grown on agar. Five of the 11 isolates tested positive in both the patch and CF assays with *A. tumefaciens* NTL4(pZLR4), indicating that these isolates produced medium- to long-chain AHLs when growing in planktonic or benthic form. Two isolates were positive only for the *A. tumefaciens* NTL4(pZLR4) patch tests.

**Table 4:** Reporter assay results and sequencing data for the eleven bacterial isolates that tested positive for AHL production.

	*Chromobacterium violaceum*CV026 Assay^1^		*Agrobacterium tumefaciens*NTL4(pZLR4) Assay^1^				
BBD andCoral Isolates	Patch Test	CF Test	Patch Test	CF Assay	GenBankAccession No.	SequenceLength (bp)	Closest Relative in GenBank (% Similarity),Accession No.
BBD-FTL-6j	**+**	−	−	**+**	KF148598	1393	2 *Vibrio rotiferianus* strain : LMG 21460 (99%), NR042081
BBD-FTL-8c	**+**	−	−	**+**	KF148599	1415	2 *Vibrio rotiferianus* strain : LMG 21460 (99%), NR042081
BBD-FLK-1d	−	−	**+**	**+**	KF148596	1326	*Nautella italica* strain : LMG 24365 (99%), NR042673
BBD-CUR-3M8	**+**	NT^3^	NT^4^	NT^4^	KF 494427	1391	*Vibrio harveyi* strain : NB0903 (99%), HM008704
BBD-CUR-3S11	**+**	NT^3^	NT^4^	NT^4^	KF 494426	1396	*Vibrio rotiferianus* strain : DB1 (99%), KC756840
BSML-FTL-6w	−	−	**+**	−	KF148595	1326	*Ruegeria scottomollicae* : LMG 24367 (96%), NR042675
BSML-FTL-7l	ND^5^	−	**+**	−	KF148600	1385	*Pseudoalteromonas phenolica* strain O-BC30 (98%), NR028809
HSML-FTL-9c	−	−	**+**	**+**	KF148601	1414	*Vibrio harveyi* strain NCIMB 1280 (99%), NR043165
HSML-FTL-9e	−	−	**+**	**+**	KF148602	1398	*Aliagarivorans marinus* strain AAM1 (95%), NR044585
HSML-FTL-9i	−	−	**+**	**+**	KF148603	1345	*Pseudoruegeria aquimaris* strain SW-255 (96%), NR043932
HSML-FTL-10a	ND^5^	−	**+**	**+**	KF148597	1387	*Pseudoalteromonas luteoviolacea* strain NCIMB 1893 (99%), NR026221

1 “+” indicates a positive assay result and a “−” indicates a negative result.

2 Although BBD-FTL-6j and BBD-FTL-8c shared the same closest relative in GenBank, these isolates showed differing colony morphologies and were isolated from two different BBD samples on three separate coral colonies.

3 Culture fluids of isolates not tested with the *C. violaceum* CV026 reporter strain.

4 Isolates not tested with the *A. tumefaciens* NTL4(pZLR4) reporter strain.

5 Not detectable. These isolates inhibited growth of the *C. violaceum* CV026 reporter strain in the patch tests, eliciting an area of clear, cell-free agar in the assay plate surrounding the bacterial patch. Additionally, isolate HSML-FTL-10a produced a dark purple pigment.

Of the 11 AHL-positive isolates, five were from BBD samples (collected from five separate BBD-infected coral colonies), two from BSML samples, and four from HSML samples. None of the eight BBD cyanobacterial strains tested positive in either AHL reporter stain assay.

### AI-2 Production

**Figure 2:**
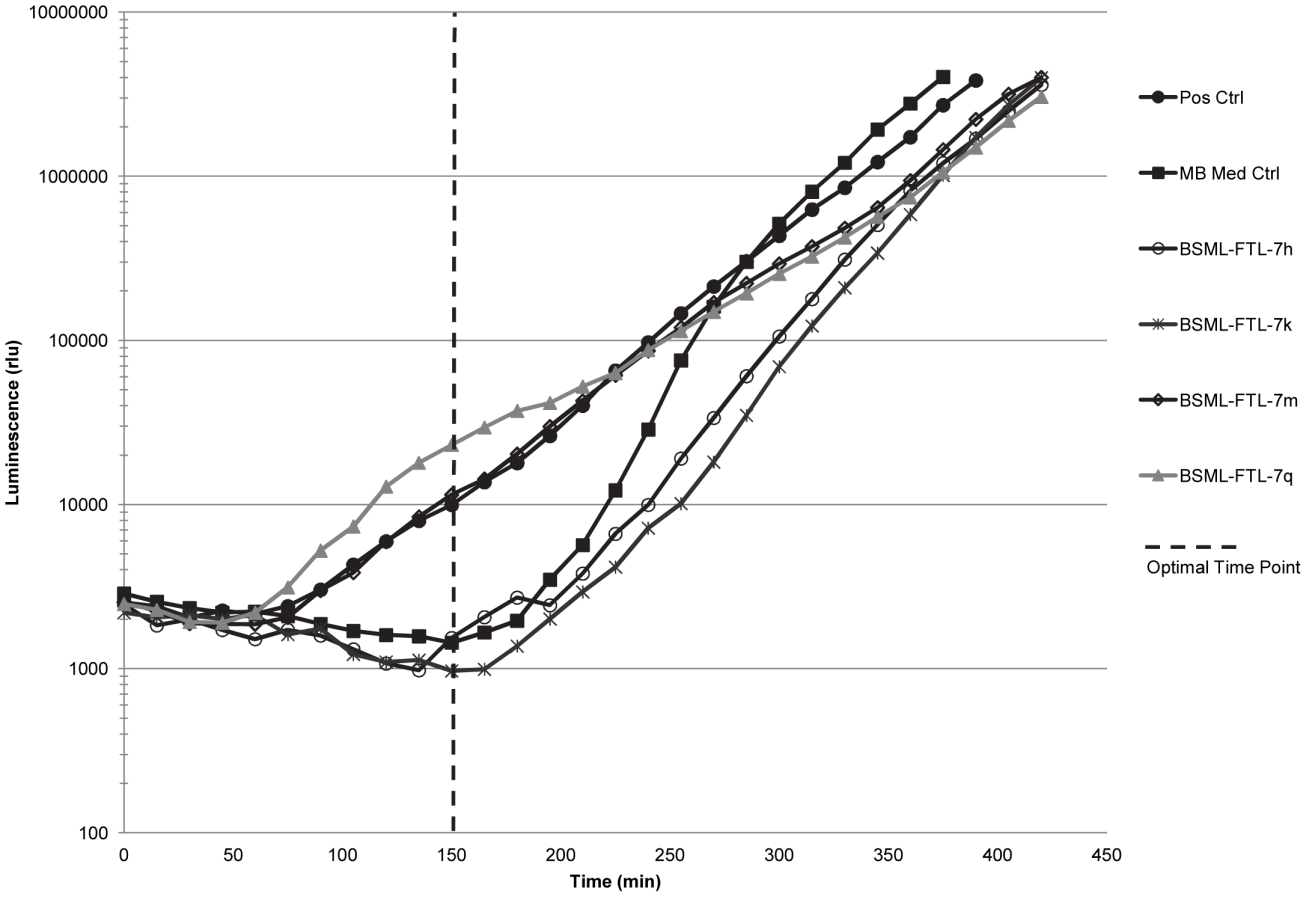
Representative experiment showing light production over time in the AI-2 reporter strain assay. The curves generated by the cell-free culture filtrates (CFs) from BSML-FTL-7m and BSML-FTL-7q (both positive in the assay) follow the positive control curve. The curves generated by the CFs from BSML-FTL-7h and BSML-FTL-7k (both negative in the assay) follow the Marine Broth (MB) medium control curve. Dashed vertical line indicates the optimal time point (OTP) of the assay, which is the time point immediately preceding self-induction by the *V. harveyi* BB170 reporter strain and the time when the mean luminescence of the medium control wells was lowest during the course of the assay. Data at the OTP is used to calculate the percentage of AI-2 (% AI-2) activity and the induction of luminescence over the positive control.

More than half (84, or 55%) of the 153 CFs tested positive in the AI-2 reporter strain assay (summarized in Table 5). Figure 2 shows results of a representative experiment using the CFs of four isolates, including two that tested positive (BSML-FTL-7m and BSML-FTL-7q) and two that tested negative (BSML-FTL-7h and BSML-FTL-7k). The positive results indicate that isolates produced DPD-like molecules (or a CAI-1 signal specific to certain *Vibrio* spp.) that can be detected by the *V. harveyi* BB170 reporter strain used in this study. The AI-2 activity induced by CFs from the positive isolates ranged widely, from approximately 28% to 346% of the positive control luminescence (Table S1) with the triplicate readings within 30% of the mean luminescence values, expressed in relative light units (rlu). The induction of luminescence over the positive control was calculated for each CF tested and ranged from 0.22–2.8 fold. 70% of the BBD CFs, 68% of the BSML CFs and 33% of the HSML CFs tested positive for AI-2 production (Table 5). None of the cyanobacterial CFs tested positive in the AI-2 assay.

**Table 5:** Autoinducer-2 activity detected using the *Vibrio harveyi* BB170 reporter strain presented according to isolate type.

Isolate Type	No. CFs Tested	No. and % Positive CFs	Percentage of Total Positive Results
BBD	63	44 (70%)	52%
BSML	37	25 (68%)	30%
HSML	45	15 (33%)	18%
Cyanobacteria	8	0	0%
**Totals**	**153**	**84 (55%)**	**100%**

**Figure 3:**
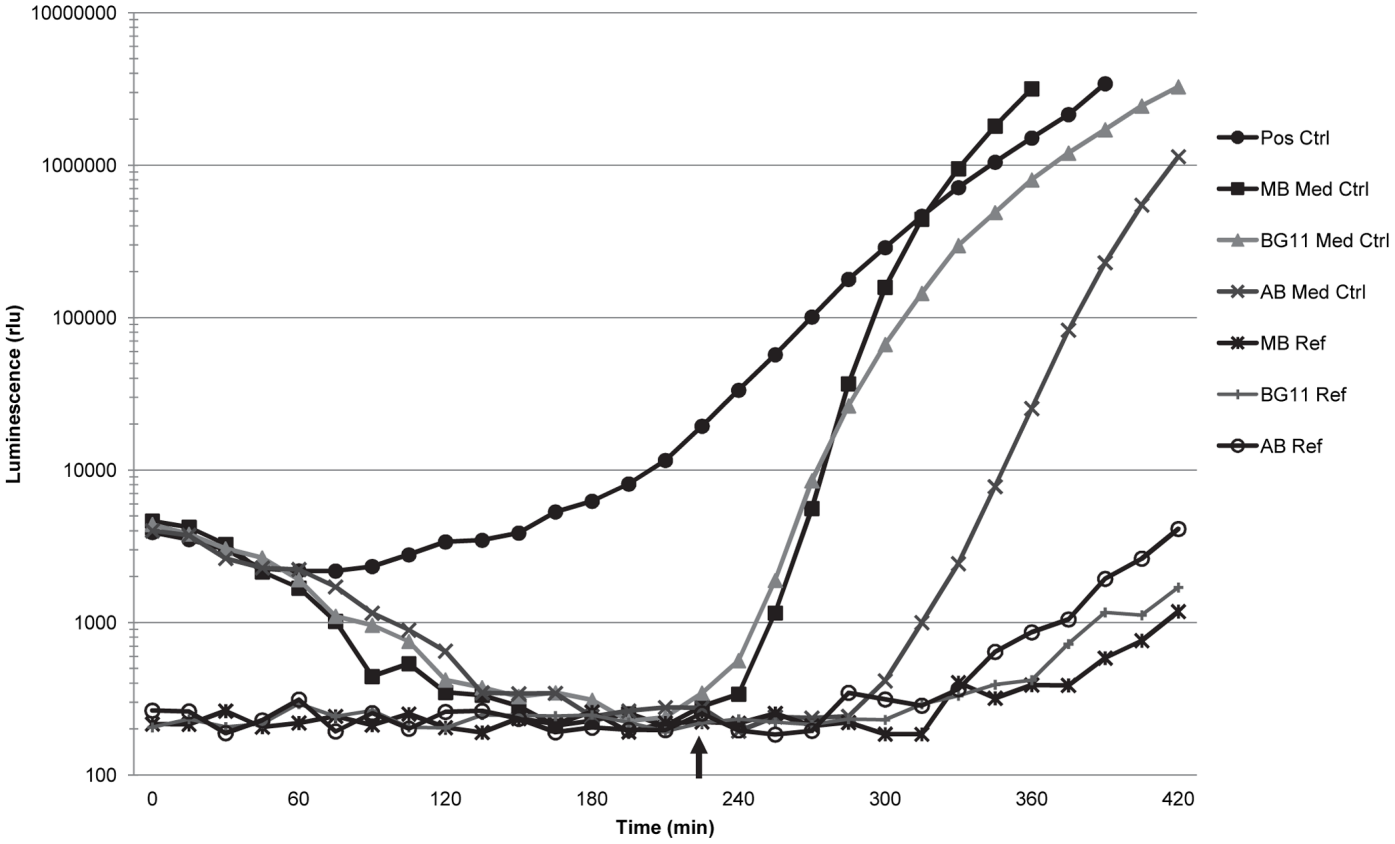
Light production of the media control wells and media reference wells in the AI-2 reporter assay. The medium control curves indicate that the three growth media used in this study did not stimulate light production prior to self-induction by the *Vibrio harveyi* BB170 reporter strain. The luminescence of the medium reference wells, which contained sterile growth media, remained minimal over the course of the AI-2 reporter assay, although some minor increases in light were measured in these wells at the end of the assay due to light contamination from adjacent wells. Arrow indicates time of self-induction by the reporter strain, after which the luminescence of the reporter strain increases rapidly.

The media used in this study did not in any case stimulate light production in the AI-2 assay prior to self-induction by the *V. harveyi* BB170 reporter strain (example shown in Figure 3). The slight rise in luminescence near the end of the experiment depicted in Figure 3 is likely the result of light contamination from adjacent wells.

### AHL Identification and Quantitation

With one exception (BSML-FTL-7l), each of the 11 isolates that were examined for identification of AHLs by LC-MS/MS produced AHLs under both planktonic (MB) and benthic (MA) conditions (Table 6), a result that is in contrast to the variable results obtained using the reporter strains. Note that of these 11 strains, 10 were discussed previously in terms of reporter strain results (see Table 4). One strain (HSML-FTL-10a) was not analyzed by LC-MS/MS because this strain failed to grow at the time of the LC-MS/MS analysis. Also note that Table 6 includes an additional isolate (BBD-FLK-1M2) not previously discussed. This isolate tested negative for AHL production using the reporter assays (thus, it is not included in Table 4); however, it is the subject of current physiological studies of BBD bacteria (data not included here), and thus was included in the LC-MS/MS analysis.

**Table 6:** AHLs detected via LC-MS/MS after 24 hour incubation.

Isolate	AHLs Detected in Liquid Culture^1^^,^ 3 ^,^ 4	AHLs Detected on Agar^2^^,^ 3 ^,^ 4	DPD Conc (µM)^1^^,^ 5
BBD-FTL-6j	C6 (78%), 3OC4 (13%), 3 others (9%)	C6 (74%), 3OC4 (11%) 3 others (15%)	0.99±0.32
BBD-FTL-8c	C6 (70%), 3OC4 (11%), 6 others (9%)	C6 (50%), 3OC6 (21%), C4 (13%), 4 others (16%)	0.94±0.09
BBD-FLK-1d	C6 (69%), 3OHC10 (9%), 9 others (22%)	C6 (61%), 7 others (39%)	1.08±0.28
BBD-CUR-3M8	C6 (92%), 3 others (8%)	C6 (69%), 3OC4 (31%)	1.10±0.49
BBD-CUR-3S11	C6 (86%), 3 others (14%)	C6 (80%), 3 others (20%)	1.38±0.19
BSML-FTL-6w	C14 (45%), C10 (26%), 3OHC4 (23%) 3OC20 (5%)	C10 (39%), 3OHC8 (23%), 3OHC4 (18%), 3 others (20%)	0.32±0.13
BSML-FTL-7l	26 AHLs measured (ranging from 0.6–14%)	ND^6^	0.48±0.09
HSML-FTL-9c	3OC4 (52%), C14(31%), 3OC18∶1(12%), 3OC20 (5%)	3OHC9∶1 (79%), 3OHC4 (21%)	1.41±0.16
HSML-FTL-9e	3OHC10 (75%), 7 others (15%)	C6 (65%), 3OHC10 (22%), 4 others (13%)	0.37±0.02
HSML-FTL-9i	3OHC10 (70%), 3OHC18 (14%), 2 others (16%)	C6 (51%), 3OHC10 (36%), 3OC4∶1 (14%)	0.31±0.08
BBD-FLK-1M2^7^	3OC6 (29%), 3OC18∶1 (19%), C14 (19%), 3OHC10 (12%), 7 others (21%)	3OHC10 (100%)	1.19±0.12

1 Results are an average of four samples (two cultures sampled twice).

2 Results are an average of six samples (three cultures injected twice).

3 Percentages represent relative abundance within the culture.

4 AHLs are comprised of a homoserine lactone ring attached to an acyl side chain (generally between 4–20 carbons in length) and may have a keto or hydroxy substituent at the C3-position. Abbreviations: C*X* = AHL contains “*X*” carbon molecules in the acyl chain; 3OC*X* – AHL has a keto substituent at the C3-position; 3OHC*X* – AHL has a hydroxyl substituent at the C3-position.

5 Error is reported at standard deviation.

6 Not detected.

7 Isolate BBD-FLK-1M2 was identified through 16S rRNA gene sequencing as a 100% match to *Ferrimonas* sp. EF3B-B688 (Accession No. KC545309.1). This isolate tested negative in the *Chromobacterium violaceum* CV026 patch test and was not tested using the *Agrobacterium tumefaciens* NTL4(pZLR4) assay.

The most common AHL variant produced among the 11 isolates was C6-HSL, produced by five (45%) of the strains in liquid culture and seven (64%) of the strains grown on plates (Table 6). This signal accounted for 50–92% of the AHLs produced (per strain) and was produced by all of the AHL-positive strains isolated from BBD and two strains from the SML of (two) apparently healthy corals (HSML samples). The second most common AHL was 3OC4-HSL, detected in five (45%) of the strains (isolated from 3 BBD, 1 BSML, and 1 HSML sample). This variant accounted for a maximum of 52% of AHL detected for strain HSML-FTL-9c (range of 11–52%). Additional AHLs were produced by fewer strains, but in some cases were the majority of the variant detected (e.g., for strains HSML-FTL-9e and HSML-FTL-9i grown in broth culture, 3OHC10-HSL accounted for 75% and 70% of AHLs produced, respectively). Other AHLs were produced in smaller amounts (see Table 6), including 3OC20-HSL which constituted 5% of AHLs produced by two isolates. To our knowledge, this AHL has not been reported in the literature.

### DPD Quantitation

LC-MS/MS was also used to assess the presence and concentration of DPD produced by the 11 isolates discussed above. As seen in Table 6, all of the isolates produced DPD at concentrations that ranged from 0.31 to 1.41 µM. All six of the BBD isolates produced high amounts of DPD (average of 1.11 µM) while, with one exception, the SML isolates (both HSML and BSML) produced much less (approximately one third to one half of the amount produced by BBD isolates). The anomalous isolate (HSML-FTL-9c), which produced the highest concentration of DPD detected for any strain independent of source, was a 99% match to *Vibrio harveyi*, a well-known AI-2 signaling species. The other five isolates produced an average of 0.37 µM DPD.

### Growth Challenge Assays

Of the 342 growth challenges, 92 (27%) resulted in significant (see methods) inhibition and eight (2.3%) resulted in significant stimulation of isolate growth (Table 7). When considered by isolate source and the number of isolates tested per source, the CFs (culture filtrates) of HSML isolates inhibited the most growth at 32% of challenges (29 of 90 tests), followed by the CFs of BSML isolates (26%, or 28 of 108), and CFs of BBD isolates (24%, or 35 of 144). Growth stimulation by CFs occurred for approximately 4% of challenges with BSML isolates, 2% of BBD isolates, and 1% of HSML isolates (Table 7). Independent group t-tests revealed that there was no significant difference (p>0.05) between the numbers of cultures inhibited or stimulated by CFs from any combination of the three isolate types.

**Table 7:** Isolates that elicited inhibition and stimulation of growth according to isolate source and production of AHLs or AI-2 activity based on reporter strain data and DPD (LC-MS/MS).

Isolate Source	CF Tested	AHL	AI-2	DPD	No. Cultures Inhibited	No. Cultures Stimulated
**BBD (144 challenges)**	BBD-FTL-6j	**+**	**+**	**+**	6	0
	BBD-FTL-8c	**+**	**+**	**+**	3	0
	BBD-FLK-1d	**+**	−	**+**	3	0
	BBD-FTL-1h^1^	−	**+**	NT	4	0
	BBD-FTL-6d^1^	−	−	NT	6	1
	BBD-FTL-6n^1^	−	**+**	NT	5	0
	BBD-FTL-6p^1^	−	**+**	NT	6	1
	BBD-FLK-1e^1^	−	**+**	NT	2	1
	**N and % of Challenges** 2				**35 (24%)**	**3 (2.1%)**
**BSML (108 challenges)**	BSML-FTL-6w	**+**	**+**	**+**	3	1
	BSML-FTL-7l	**+**	−	**+**	9	0
	BSML-FTL-6u^1^	−	**+**	NT	3	1
	BSML-FTL-7d^1^	−	**+**	NT	5	2
	BSML-FTL-7q^1^	−	**+**	NT	4	0
	BSML-FLK-1d^1^	−	**+**	NT	4	0
	**N and % of Challenges** 2				**28 (26%)**	**4 (3.7%)**
**HSML (90 challenges)**	HSML-FTL-9c	**+**	**+**	**+**	3	0
	HSML-FTL-9e	**+**	−	**+**	4	0
	HSML-FTL-9i	**+**	**+**	**+**	4	0
	HSML-FTL-10a^1^	**+**	−	NT	11	0
	**HSML-FTL-10r** 1	−	**+**	**NT**	**7**	**1**
	**N and % of Challenges** 2				**29 (32%)**	**1 (1.1%)**
**Total Challenges (342)**					**92 (27%)**	**8(2.3%)**

1 Not tested. These strains were not analyzed for DPD production using LC-MS/MS (refer to Table 6).

2 The percent value here is the percent of the total challenges (144 BBD strains, 108 BSML strains, and 90 HSML strains) that resulted in inhibition or stimulation of growth in each isolate source category.

The 19 isolates examined in the growth challenges included nine AHL-positive isolates (Table 7). Five of these nine also tested positive for AI-2 activity using the *V. harveyi* BB170 reporter strain assay, and eight produced DPD detected by LC-MS/MS (Table 6). Of the 10 AHL-negative isolates, nine tested positive for AI-2 activity. Table 8 examines the correlation between the presence/absence of AHL, AI-2 activity, and DPD among isolates that caused inhibition of growth. Strain HSML-FTL-10a (which inhibited seven strains and stimulated one; Table 7) was not included in this data set because it did not grow during the LC-MS/MS experiments. Although HSML-FTL-10a was negative for AI-2 activity using the reporter strain, three additional strains that also tested negative for the reporter strain assay produced detectable DPD (Table 6), thus we cannot rule out that HSML-FTL-10a also produces DPD. The resulting data set therefore consists of 81 challenges (using 18 isolates) that resulted in inhibition of growth. Of these, nine strains produced AI-2/DPD (positive for at least one of these assays) but no AHL, and were responsible for 49% of the 81 cases of growth inhibition. Eight strains produced both AI-2/DPD and AHL, and inhibited 43% of the 81 cases. One strain (BBD-FTL-6d) did not produce AI-2/DPD or AHL and inhibited six isolates. There was no statistically significant difference (independent group t-tests, p>0.05) between the number of cultures inhibited by isolates producing AI-2/DPD only and those that produced both AHL and AI-2-DPD.

**Table 8:** Correlations between autoinducer production and inhibition of growth.

Source of Isolate and Autoinducer Produced	No. Isolates that Elicited Inhibition (No. Test Strains Inhibited)			% of Inhibition^1^
Source of Isolate	BBD	BSML	HSML	
AI-2/DPD	4 (17)	4 (16)	1 (7)	49.4
AHL and AI-2/DPD	3 (12)	2 (12)	3 (11)	43.2
Neither	1 (6)	0	0	7.4
Total	8 (35)	6 (28)	4 (18)	100%

1 Percent of 81 assays that resulted in growth inhibition. Isolate HSML-FTL-10a not included (see text).

## Discussion

### Quorum Sensing Signals in BBD and Coral-Associated Bacteria

This study revealed the presence of AHLs in nine freshly collected BBD microbial mat samples from two Caribbean coral species. In each case, the full BBD community was present. The immediate questions that rise from these results are: 1) what microbial constituents within this pathogenic polymicrobial community are synthesizing AHLs?; 2) are the AHL producers in BBD also present in the coral microbiome?; 3) what are the structures of the AHLs produced?; and 4) do BBD and SML bacteria also produce AI-2? We addressed each of these questions in the present study.

The observation that all (full community) BBD samples examined using the AHL reporter strains were positive for the presence of AHLs is in agreement with previous studies on QS signal production in cyanobacterial-dominated communities in other environments. These include *Trichodesmium* colonies in the North Atlantic [82], marine stromatolites in the Bahamas [83], and cyanobacterial mats in Swiss alpine wetland ponds [84]. On the other hand, although cyanobacterial isolates have been reported to produce AHLs in the laboratory [85], none of the eight BBD cyanobacterial isolates examined in this study tested positive in either AHL reporter strain assay. We note that our cyanobacterial culture medium had a pH of ∼8 (or higher during photosynthesis), and that alkaline pH has been demonstrated to degrade AHL signals [62], [64]. Furthermore, it is possible that the AHL reporter strain assays yielded false-negative results for a variety of other reasons (e.g., the AHL produced was undetectable by the reporter strains, AHL concentrations were below the detectable levels of the reporter strains, AHL production was inhibited by the laboratory conditions used in this study, the isolate was also producing quorum quenching molecules that inhibited detection of AHLs, and/or other abiotic factors besides pH degraded the AHL signal). These same scenarios could explain false negative results in any of the reporter strain assays used to detect QS signals in this study.

To investigate the identity of culturable BBD bacteria that are capable of producing AHLs (as well as DPD), we tested bacterial isolates from the complex microbial communities that constitute BBD, the apparently healthy SML of BBD-diseased colonies, and the SML of healthy colonies. We found that 11 of 199 (5.5%) isolates, all bacterial, from BBD, BSML, and HSML are able to produce QS signals under laboratory conditions. This result is similar to the 4% observed in a study [27] in which more than 300 bacterial cultures isolated from marine invertebrates (i.e., coral SML, other marine invertebrates, and dinoflagellate symbionts) were screened using a variety of reporter strains that detect AHLs of varied chain lengths. We also showed that 55% of CFs tested positive for AI-2 activity (or the CAI-1 signal specific to some *Vibrio* spp.), as detected by the *V. harveyi* BB170 reporter strain. The high percentage of isolates capable of AI-2 activity is not unexpected, as this molecule is considered to be an interspecies signal molecule [46], [86]. It should be noted that AHL and AI-2 production in some bacteria may be specific to a certain portion of the bacterial growth phase [87], [88]. For the bacterial isolates investigated in this study, all CFs were collected during early stationary phase. Thus, an isolate producing QS signals during a different growth phase may have produced a negative result in the reporter strain assays. Based on subsequent 16S rRNA gene sequencing, all 11 AHL-producing isolates most closely aligned with members of the *Alpha-* and *Gamma-proteobacteria* (Table 4). Five of these were closely related to *Vibrio* spp. *Vibrio fischeri* and *V. harveyi* represent the archetypal system for AHL- and AI-2-regulated gene expression [33], [89], and a variety of *Vibrio* spp. have been documented to produce AHLs [90], [91], [92]. *Vibrios* have been routinely detected in BBD [42], [93], [94], and it has been suggested that proteolytic activity by *Vibrio* spp. might play a role in BBD pathology [93]. *Vibrio* strains isolated from both healthy and diseased corals have also been shown to produce AHLs [54].

Two AHL-producing isolates (BBD-FLK-1d and BSML-FTL-6w) were most closely related to strains belonging to the *Rhodobacteraceae*, also commonly detected in BBD and coral SML communities [42], [43]. AHL production by members of the *Roseobacter-Ruegeria* subgroup of the *Alpha-proteobacteria* has been previously demonstrated [95], [96], [97]. Isolates BSML-FTL-7l and HSML-FTL-10a were most closely related to the genus *Pseudoalteromonas*, also previously documented to produce AHLs [98], [99]. Isolate HSML-FTL-10a was most closely related to *P. luteoviolacea* strain NCIMB 1893 (99% similarity) and, consistent with the description of this strain as a producer of violacein [100], isolate HSML-FTL-10a produced a dark purple pigment. *P. luteoviolacea* has been documented to have antibacterial activity [100], and antibiotic activity has also been demonstrated for violacein [101].

### QS Signals in the BBD Mat and Coral SML

Within the BBD mat, an exopolysaccharide (EPS) matrix surrounds the diverse members of the BBD microbial community. High concentrations (>600 µM) of AHLs have been measured within *Pseudomonas aeruginosa* biofilms [102], suggesting that bacterial EPS may effectively sequester QS signals. Because the surface of the BBD mat is exposed to seawater, and thus diffusion, advection, and dilution [103], accumulation of QS signals would likely be greater in the depths of the BBD mat than on the mat surface. In a similar manner, coral mucus forms a protective coating over the coral surface and is present as a thin layer that can be considered as a biofilm. While the chemical composition of coral mucus varies between species, the basic structure of the SML consists of an insoluble, hydrated glycoprotein [11], [104]. The gel-like nature of coral mucus would likely concentrate particularly hydrophobic signal molecules similar to EPS.

Within BBD, the persistence of QS signals may be impacted by the local chemical and physical microenvironment to a greater extent than within non-cyanobacterial associated biofilms. Alkaline conditions may result in hydrolysis of the AHL lactone ring [62], [64] with short-chain (< C10) AHLs appearing to be more susceptible [64], [83]. The BBD mat contains pronounced biogeochemical microgradients of oxygen, pH, and sulfide [22], [23] which fluctuate based on changes in the predominant metabolic pathways occurring within the BBD mat. During daylight, when oxygenic photosynthesis dominates, the pH in the BBD mat ranges from 7.58–8.13 [23], while in darkness, when predominant metabolic pathways shift to respiration and fermentation, pH values drop to 7.33–7.49 [23]. Based on the relative stability of AHLs in terms of pH [83], [105], microbial mats in general could use both short- and longer-chain AHL signaling during the night and longer-chain AHL signaling during the day. Coral mucus itself is acidic (pH of 5.5 to 7.7) in comparison to surrounding seawater [106]; thus, AHL signals within the coral SML would likely remain stable enough for signaling to occur both night and day. We found both short- and long-chain AHLs in this study, including variant 3OC20-HSL produced by two isolates. To our knowledge this AHL has not been reported in the literature.

The implications of abiotic and biotic influences on AI-2 signaling and the impact of the natural environment are not well understood at this time. Like AHLs, production of DPD may be dependent on the particular strain, the growth phase, or the culture medium [88], [107]. DPD appears to be relatively stable in comparison to the AHLs [82]. We found that 70% of the CFs from BBD isolates and 68% of the CFs from BSML isolates tested positive for AI-2 activity, compared to only 33% of the CFs from HSML isolates. All of the 11 isolates we examined for DPD production produced detectable amounts of this precursor to AI-2, with BBD isolates producing two to three times as much as the SML isolates (Table 6). It is possible that AI-2 signaling may be occurring more frequently within the BBD microbial consortium as compared to the coral SML, if AI-2 signaling is indeed occurring within these environments.

### Interactions among Isolates from BBD and SML

Results of the growth challenges revealed that 27% of 342 challenges resulted in significant inhibition of bacterial growth. While others have detected increased antimicrobial activity by the mucus of healthy corals, as opposed to diseased corals [28], [29], this study found no significant difference in antimicrobial production based on isolate source (BBD, BSML, or HSML). Toxins have been documented previously to be produced by BBD cyanobacteria [15], [16], [17], [18] and antimicrobials by coral-associated bacteria [29], [30], [108], [109], and it is likely that antimicrobial production contributes to the temporal and spatial dynamics occurring in BBD [110]. Interestingly, only 2% of the 342 growth challenges exhibited significant growth stimulation by the culture filtrates. The vast majority (∼88%) of these stimulatory CFs were from BBD isolates. Thus, cooperation between microbes may be more common in interactions among members of the BBD than among SML bacteria.

Almost all (∼93% of cases) of the growth inhibition documented in the challenge study was caused by the CFs of isolates that were positive for AI-2 activity and/or production of DPD. AI-2 is widely recognized for its involvement in interspecies signaling and has been linked to the upregulation of antibiotic biosynthesis [50], [51]. Regulation of bacterial relationships within complex microbial communities is another proposed role of DPD in the natural environment [35]. Our findings are in complete agreement with this proposal. For example, isolates that produced AI-2/DPD (with some also producing AHLs) were cultured from BBD, BSML, and HSML, and were shown to inhibit growth of isolates from all three of these sources. Thus, if present and active within these natural environments, it is possible that AI-2 could regulate both pathogenic and probiotic microbial populations with communication between such populations. Only one strain that caused growth inhibition produced neither AI-2/DPD nor AHL. Further research is needed to determine if this is a pattern that holds true for bacterial members of complex communities in general.

### Potential Roles of QS Signal Production in Coral Health and Disease

This is the first report of the presence of AHLs in samples freshly collected from *in situ* coral disease. Only one previous study [54] has directly and empirically addressed the potential role of QS in coral health and disease. Similar to our study, Tait *et al*. [54] used reporter strains (as well as thin layer chromatography) to detect AHLs and AI-2 production by bacteria (*Vibrio* spp. and *Photobacterium rosenbergii* strains) isolated from or near corals. Twenty-nine isolates were tested, of which 17 produced AHLs and 29 (100%) produced AI-2. Six of the 29 strains were isolated from the tissue of “diseased” corals, although the disease was not identified; of these, three were positive for AHL production. Three strains were isolated from bleached corals (two from tissue, one from mucus), with both tissue samples positive for AHL production and the mucus sample negative. An additional 11 samples are identified as from the water in association with specific coral species; however, the distance of the sample from the coral surface is not provided; of these, six were positive for AHLs. Finally, of nine isolates from tissue or mucus samples associated with presumably healthy corals (only one identified as such), five (two tissue and three mucus) produced AHLs. These results [54] suggest that QS may be an active process in the coral microbiome (and coral environment), and our findings support their conclusion.

Despite nearly 40 years of research, the etiology, pathogenesis, and pathobiology of BBD remain unresolved. Mechanisms of BBD mat formation and organization, the mode of microbial recruitment to the BBD community, and the factors leading to initiation of the pathogenic, migrating band are not understood. It is, however, well known that AHLs produce and control similar virulence factors in other systems, including in laboratory experiments using the coral pathogen *Serratia marcescens* [111]. Although there has been little work on DPD production by coral-associated bacteria, AI-2 signaling is also known to be associated with disease function in pathogenic bacteria in general (reviewed in [35], [46]).

If QS is indeed occurring within BBD, the potential roles of AHL and/or AI-2 signals with regard to virulence, biofilm structure, and community composition makes QS an important aspect of future study for BBD as well as other coral diseases. QS between potential pathogens and the healthy coral microbiota may contribute to disease processes as well. Future research to elucidate the roles that QS signaling molecules play in coral health and disease will require thorough investigations targeting specific effects of QS signals on regulatory and physiological systems, as well as gene expression.

## Supporting Information

Table S1 **Percentage of AI-2 activity (± standard deviation) and induction of luminescence values for the 84 CFs that tested positive in the AI-2 assay.** The data are presented in descending order based on percentage of AI-2 activity. The induction of luminescence was calculated by dividing the sample luminescence by the positive control luminescence at the optimal time point of the assay.
(XLSX)
